# MicroRNA-204 Is Necessary for Aldosterone-Stimulated T-Type Calcium Channel Expression in Cardiomyocytes

**DOI:** 10.3390/ijms19102941

**Published:** 2018-09-27

**Authors:** Riko Koyama, Tiphaine Mannic, Jumpei Ito, Laurence Amar, Maria-Christina Zennaro, Michel Florian Rossier, Andrés Daniel Maturana

**Affiliations:** 1 Graduate School of Bioagricultural Sciences, Nagoya University, Nagoya 464-8601, Japan; kerry.1383kr@gmail.com (R.K.); jumpei.ito@kcl.ac.uk (J.I.); 2 Department of Human Protein Science, University of Geneva, CH-1211 Geneva, Switzerland; tiphaine.mannic@gmail.com; 3 Inserm, UMRS_970, Paris Cardiovascular Research Center, 75015 Paris, France; laurence.amar@aphp.fr (L.A.); maria-christina.zennaro@inserm.fr (M.-C.Z.); 4 Université Paris Descartes, Sorbonne Paris Cité, 75015 Paris, France; 5 Assistance Publique-Hôpitaux de Paris, Hôpital Européen Georges Pompidou, Unité Hypertension artérielle, 75015 Paris, France; 6 Assistance Publique-Hôpitaux de Paris, Hôpital Européen Georges Pompidou, Service de Génétique, 75015 Paris, France; 7 Central Institute of Hospitals, Hospital of Valais, CH-1951 Sion, Switzerland

**Keywords:** microRNA, T-channels, aldosterone, cardiomyocytes

## Abstract

Activation of the mineralocorticoid receptor (MR) in the heart is considered to be a cardiovascular risk factor. MR activation leads to heart hypertrophy and arrhythmia. In ventricular cardiomyocytes, aldosterone induces a profound remodeling of ion channel expression, in particular, an increase in the expression and activity of T-type voltage-gated calcium channels (T-channels). The molecular mechanisms immediately downstream from MR activation, which lead to the increased expression of T-channels and, consecutively, to an acceleration of spontaneous cell contractions in vitro, remain poorly investigated. Here, we investigated the putative role of a specific microRNA in linking MR activation to the regulation of T-channel expression and cardiomyocyte beating frequency. A screening assay identified microRNA 204 (miR-204) as one of the major upregulated microRNAs after aldosterone stimulation of isolated neonatal rat cardiomyocytes. Aldosterone significantly increased the level of miR-204, an effect blocked by the MR antagonist spironolactone. When miR-204 was overexpressed in isolated cardiomyocytes, their spontaneous beating frequency was significantly increased after 24 h, like upon aldosterone stimulation, and messenger RNAs coding T-channels (CaV3.1 and CaV3.2) were increased. Concomitantly, T-type calcium currents were significantly increased upon miR-204 overexpression. Specifically repressing the expression of miR-204 abolished the aldosterone-induced increase of CaV3.1 and CaV3.2 mRNAs, as well as T-type calcium currents. Finally, aldosterone and miR-204 overexpression were found to reduce REST-NRSF, a known transcriptional repressor of CaV3.2 T-type calcium channels. Our study thus strongly suggests that miR-204 expression stimulated by aldosterone promotes the expression of T-channels in isolated rat ventricular cardiomyocytes, and therefore, increases the frequency of the cell spontaneous contractions, presumably through the inhibition of REST-NRSF protein.

## 1. Introduction

Mineralocorticoids, in particular aldosterone, are associated with the development of cardiac hypertrophy [1] and heart failure [2]. Clinical trials have demonstrated that mineralocorticoid receptor (MR) antagonists, such as spironolactone, improve the cure rate of patients with heart failure [3]. Patients with primary aldosteronism have a thicker left ventricular wall and a higher left ventricular mass index compared to patients with essential hypertension despite both groups having similar blood pressure [4]. In addition to studies on patients, the link between hyperaldosteronism and heart failure progression has also been documented in experimental animals [5,6]. Aldosterone might therefore have a direct effect on cardiovascular tissue in addition to its hypertensive effect. Indeed, MR activation in cardiac myocytes results in the upregulation of numerous ion channels including voltage-gated Na^+^ channels [7], voltage-gated K^+^ channels [8], voltage-gated Ca^2+^ channels [9,10,11], and hyperpolarization-activated cyclic nucleotide-gated channels HCN4 [12], which promotes hypertrophy development and heart failure [8,13,14]. In particular, it has been shown that cardiac T-type voltage-gated Ca^2+^ channel expression and activity are upregulated by aldosterone in neonatal rat cardiomyocytes [10,11,15]. In the adult heart, T-type Ca^2+^ channels drive the pacemaker depolarization in the sinoatrial node [16,17,18]. T-type Ca^2+^ channel re-expression in the adult ventricle has been associated with the development of cardiac hypertrophy and heart failure [19,20].

The regulatory mechanisms of T-type Ca^2+^ channel expression by aldosterone require several glucocorticoid response elements (GREs) on the promoter of *CACNA1G* (Calcium Voltage-Gated Channel Subunit Alpha1 G), the gene encoding for CaV3.1, one of the two T-type Ca^2+^ channels expressed in the heart. Mutations of GRE1 abolish *CACNA1G* transcriptional activation induced by aldosterone stimulation [21]. However, the regulatory mechanisms of the *CACNA1H* (Calcium Voltage-Gated Channel Subunit Alpha1 H) gene which encodes CaV3.2, the other cardiac T-type Ca^2+^ channel, by aldosterone remain elusive. MicroRNAs (miRNAs) are single-stranded RNA molecules composed of about 22 non-coding nucleotides that regulate the translation of their target mRNAs [22]. The contribution of microRNA to cardiovascular development and diseases has been widely explored [23]. Conditional deletion of Dicer, which is an essential component in the miRNA biogenesis, causes sudden death, accompanied by cardiac remodeling in postnatal murine cardiomyocytes [24]. Targeted Dicer deletion in adult mice hearts provokes hypertrophy, cardiac dysfunction, and fetal gene reactivation [24]. Moreover, the expression of several miRNAs is altered in hypertrophied mice hearts [25].

The role of particular miRNAs in cardiac hypertrophy has also been investigated. Overexpression of miR-208a in the heart is sufficient to induce cardiac hypertrophic growth in mice, due to the inhibition of two hypertrophic negative regulators, thyroid hormone-associated protein 1 and myostatin [26]. The expression of miR-23a is upregulated in hypertrophied cardiomyocytes [27]. MiR-23a is regulated by NFATc3 (Nuclear Factor of Activated T Cells 3) which mediates the calcineurin hypertrophic signal and targets the anti-hypertrophic protein muscle-specific ring finger protein 1 (MuRF1) [27].

Some studies have demonstrated the interplay between ion channels and miRNAs. For example, the expression of the type II inositol 1,4,5-trisphosphate receptor (IP3RII), an intracellular calcium channel located in the ER, is regulated by miR-133a, resulting in an altered calcium signaling in hypertrophied cardiomyocytes [28]. The inhibition of miR-133a in cardiomyocytes increases IP3RII levels, hypertrophic marker gene levels, and cell surface area [28]. Defining the interplay of miRNAs and ion channels might provide important insights into the regulatory mechanisms of cardiac hypertrophy.

Given the apparent contribution of miRNAs in cardiovascular diseases, we hypothesized that specific miRNAs could mediate some effects of aldosterone in cardiomyocytes, in particular, the expression of CaV3.1 and CaV3.2 T-type calcium channels.

In an initial study, we found that aldosterone stimulates the expression of several miRNAs, with miR-204 being the most upregulated one. The overexpression of miR-204 led to a marked increase in the spontaneous beating frequencies of isolated neonatal rat cardiomyocytes, mimicking our previously reported effect of aldosterone on the rate of spontaneous contractions. Concomitantly, miR-204, by itself, stimulated an increase in T-type calcium currents and mRNA expression of both CaV3.1 and CaV3.2. In addition, the protein expression of a known repressor of CaV3.2 [29], REST-NRSF (RE1-silencing transcription factor also known as neuron-restrictive silence factor), was found to be reduced upon overexpression of miR-204. The repression of miR-204 expression led to the suppression of an aldosterone-stimulated increase in T-type calcium currents and CaV3.1 and CaV3.2 mRNA expression. Taken together, these results uncover a mechanical pathway of CaV3.2 T-type calcium channel stimulated expression by aldosterone involving the inhibition of REST-NRSF by miR-204 upregulation in rat cardiomyocytes.

## 2. Results

### 2.1. Aldosterone Stimulates miR-204 Expression

**Figure 1:**
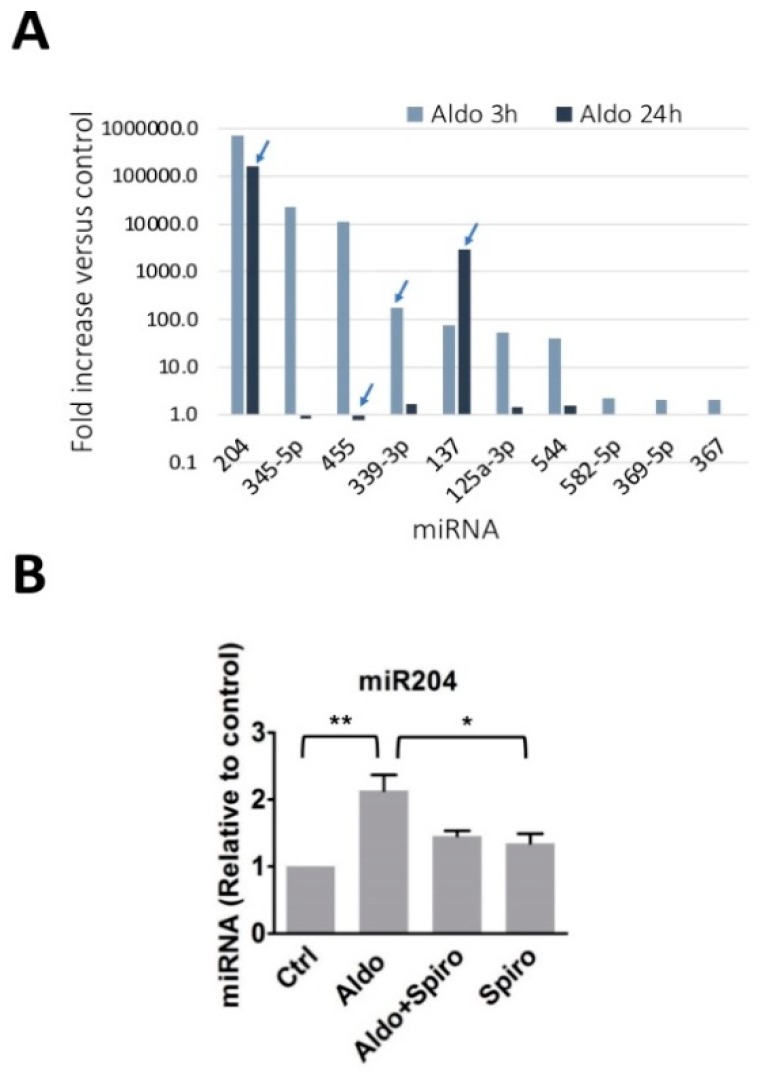
MicroRNA regulation by aldosterone in rat neonate ventricular cardiomyocytes. (**A**) Isolated ventricular cardiomyocytes were incubated for 3 h or 24 h with or without aldosterone (1000 nmol/L). Total RNA was then collected, and miRNA content was analyzed by qPCR on a Taqman low-density array (TLDA) covering more than 300 murine miRNA targets. The response to aldosterone of each miRNA was expressed as fold increase, calculated versus the control amount determined in unstimulated cells. The graph shows the mean values from two experiments performed from two independent cell preparations (the arrow indicates results differing by more than 35% from one experiment to the other). The miRNAs displaying higher signal increases in response to 3 h of exposure to aldosterone are shown. (**B**) The graph shows the relative expression of miR-204 determined in control rat neonatal ventricular cardiomyocytes, and in cells treated for 24 h with aldosterone alone (1 µM), aldosterone and spironolactone (10 µM), or spironolactone alone. The miR-204 expression levels were determined by real-time quantitative PCR using specific primers and normalized to U6 SnRNA. The bars and error bars indicate the means + SEMs (*n* = 3). ** means *p* < 0.01 vs. control, and * *p* < 0.05.

Aldosterone has been shown to upregulate T-type Ca^2+^ channel expression and activity in cardiomyocytes, as well as the cell spontaneous beating frequency [10,11]. We hypothesized that some specific miRNAs might play a role in the aldosterone-stimulated increase of T-type Ca^2+^ channel expression in ventricular cardiomyocytes. A microarray screening, allowing the simultaneous measurement of the expression levels of 2 × 381 miRNAs, was performed in naïve or aldosterone-stimulated (1 µM for 3 or 24 h) neonatal rat cardiomyocytes. A high, supra-physiological concentration of aldosterone was used throughout the present study in order to activate both MR and GR receptors [13]. The results showed that, under these conditions, the most upregulated miRNA by 3 h aldosterone was miR-204 (Figure 1A). In contrast to other miRNAs, miR-204 remained markedly elevated even after 24 h of stimulation with the hormone. We then precisely evaluated the miR-204 expression levels in aldosterone-treated cardiomyocytes by quantitative PCR. Aldosterone (1 µM for 24 h) significantly raised miR-204 expression levels in cardiomyocytes (Figure 1B). We then verified that the effect of aldosterone on miR-204 is specifically mediated by the activation of the mineralocorticoid receptor (MR). Spironolactone (10 µM for 24 h), a strong MR antagonist, prevented miR-204 upregulation by aldosterone in cardiomyocytes (Figure 1B).

These results, therefore, suggest that aldosterone stimulates an increase in miR204 expression in cardiomyocytes via MR activation.

### 2.2. Chronotropic Action of miR-204 in Isolated Cardiomyocytes

**Figure 2:**
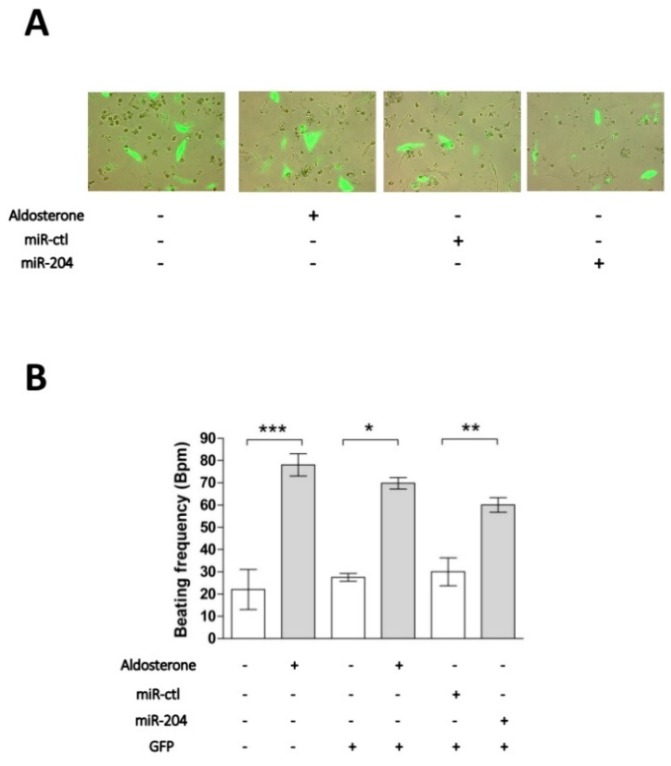
MiR-204 mimics the chronotropic action of aldosterone in cardiomyocytes. (**A**) Neonate rat ventricular cardiomyocytes in culture were electroporated and immediately transfected with a plasmid coding for GFP either alone or in the presence of miR-204 or a control microRNA (miR-ctl). Images show representative cell fluorescence observed 24 h after cell transfection under various experimental conditions. (**B**) The cardiomyocyte beating frequency was determined by counting individual cell contractions under the microscope 24 h after cell transfection and/or incubation under several experimental conditions (indicated below the graph by the presence of a + sign). When cells were co-transfected with the GFP plasmid, only the beating frequency of fluorescent cells was determined. Data are means ± SEMs calculated from two independent cell preparations in which myocyte beating frequency was determined in 8 to 16 different fields for each condition. The statistical significance of the difference versus respective controls (* *p* < 0.05, ** *p* < 0.01, and *** *p* < 0.001) was assessed by unpaired Student’s *t*-tests.

We previously showed that aldosterone stimulation of isolated cardiomyocytes results in a marked increase of their spontaneous beating frequencies [10]. We thus tested whether miR-204 overexpression can mimic the effect of aldosterone after 24 h of transfection (Figure 2). As previously reported, stimulation of 1 µM aldosterone for 24 h significantly increases the spontaneous beating rate of cardiomyocytes as compared to the control condition. Neither the expression of GFP alone, nor GFP with a control miRNA, affected the beating rate. However, miR-204 overexpression significantly increased the beating rate of cardiomyocytes (Figure 2B). In these experiments, the presence of a plasmid coding for GFP allowed us to select only transfected cells; indeed, only green fluorescent cells were considered for measuring the beating frequency (Figure 2A). These results suggest that miR-204 plays a role in the chronotropic effect of aldosterone in cardiomyocytes.

### 2.3. T-Type Ca^2+^ Channel Expression in miR-204-Overexpressing Cardiomyocytes

**Figure 3:**
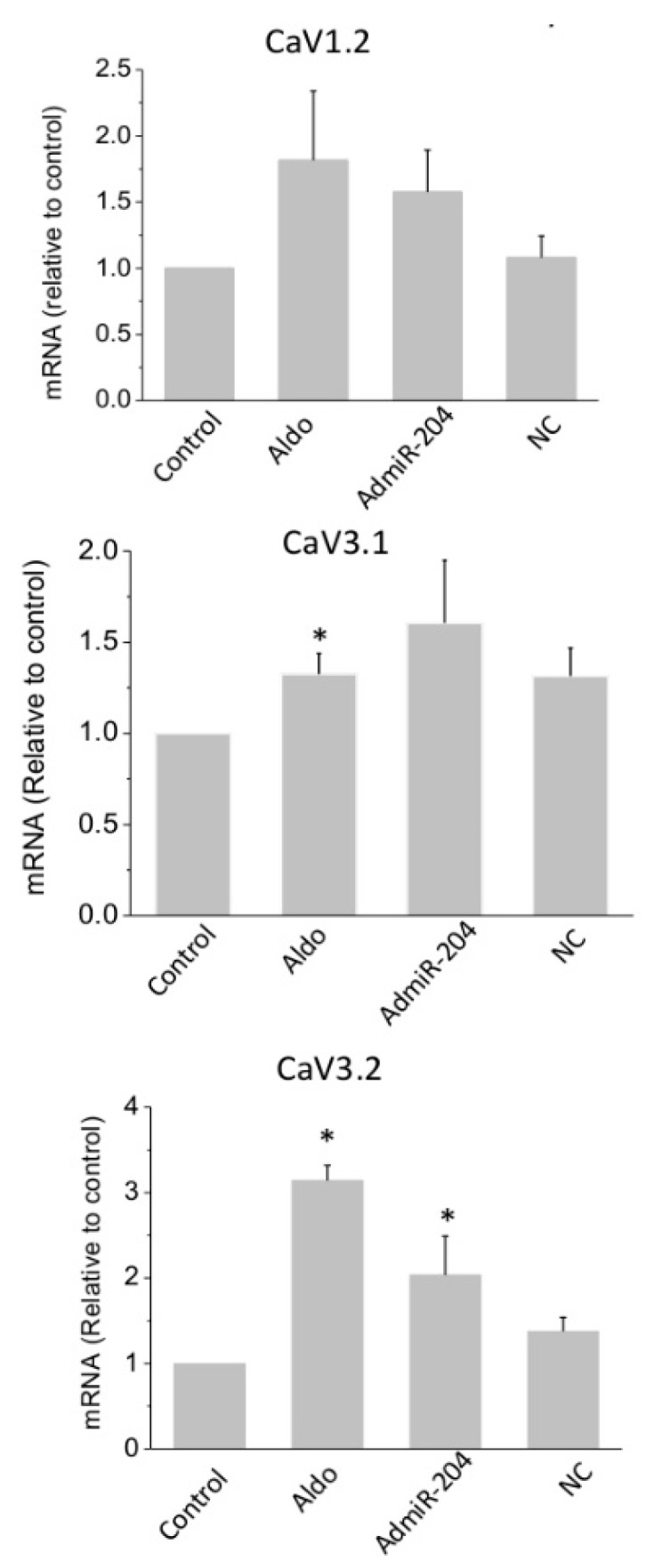
Overexpression of miR-204 increases the expression of calcium channel alpha1 subunits. The mRNA levels for L-type and T-type Ca^2+^ channel pore subunits CaV1.2, CaV3.1, and CaV3.2 was determined in control cells and in aldosterone (aldo, 1 µM for 24 h)-treated, miR-204 adenovirus-infected (AdmiR-204), or shLuc adenovirus-infected cardiomyocytes (NC). Bars and error bars indicate the means + SEMs (*n* = 3). * means *p* < 0.05 vs. control.

We next tested whether miR-204 overexpression can mimic the effect of aldosterone on T-type Ca^2+^ channel expression and activity. Overexpression of miR-204 was achieved by adenoviral transduction (Figure S1). The overexpression of miR-204 for 24 h resulted in a significant increase in CaV3.2 mRNA levels (Figure 3). The expression of the pore subunit of the T-type, CaV3.1, and L-type, CaV1.2, voltage-gated calcium channels were also affected by miR-204, but the effect in these cases remained below statistical significance. While the responses to aldosterone and miR-204 were quite similar on CaV1.2 and CaV3.1 expression, the hormone was much more efficient at inducing an elevation of CaV3.2, suggesting that aldosterone recruits additional mechanisms for increasing the expression of the latter channel.

**Figure 4:**
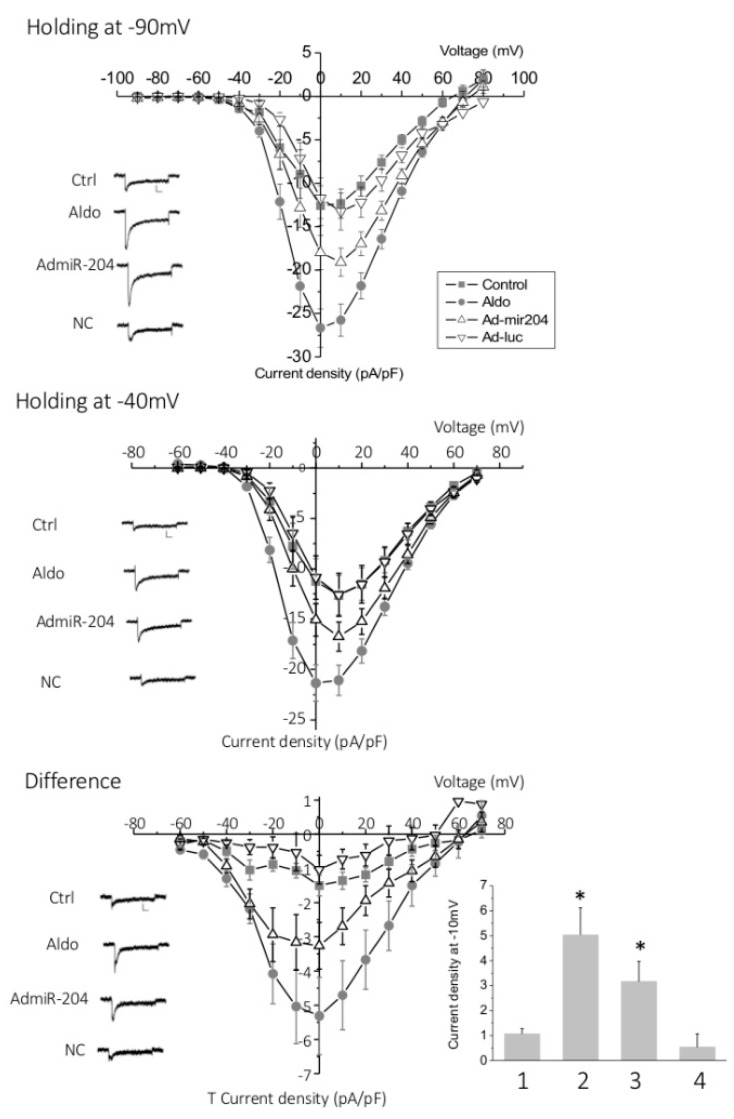
T-type and L-type Ca^2+^ currents are increased upon miR-204 overexpression. The graphs show the current density-voltage relationship obtained from whole cell patch clamp experiments in control (Ctrl), aldosterone (1 µM for 24 h)-treated (Aldo), miR-204 adenovirus-infected (Admir-204), and luc-adenovirus-infected cardiomyocytes (Ad-luc) obtained from holding potential at −90 mV (upper panel), reflecting the total (L-type and T-type) calcium current, or at −40 mV (middle panel), reflecting L-type current. The difference plots, reflecting T-type Ca^2+^ currents, are shown in the lower panel. Traces are representative examples of current recordings at –20 mV depolarization steps. Scale bars are 100 pA (vertical) and 25 ms (horizontal). Data are means ± SEMs (*n* = 11–16). The bar graph represents the means + SEMs of the T current density recorded at −10 mV in control (**1**), aldosterone (1 µM for 24 h)-treated (**2**), miR-204 adenovirus-infected (**3**), and luc-adenovirus-infected (**4**) cardiomyocytes (* *p* < 0.05).

Next, T-type Ca^2+^ current densities were determined by analyzing the difference between total Ca^2+^ currents measured from a holding potential at −90 mV and L-type specific Ca^2+^ currents evoked from a holding potential at −40 mV in the whole cell configuration of the patch clamp technique. Aldosterone treatment (1 µM for 24 h) significantly and markedly increased both T-type and L-type Ca^2+^ currents between −20 mV and +10 mV (Figure 4). The overexpression of miRNA-204 partially mimicked the effect of aldosterone, while cells infected with the luciferase gene displayed currents similar to those measured in control, naïve cells.

Taken together, these results suggest that miR-204 increases the expression of the L-type channel subunit CaV1.2, as well as both of the T-type channel subunits, CaV3.1 and CaV3.2, downstream of MR activation in neonatal rat cardiomyocytes. They also suggest that Ca^2+^ channel regulation in response to aldosterone requires a not yet identified miR-204 target protein that is expected to downregulate Ca^2+^ channel expression.

### 2.4. MicroRNA-204 Is Necessary for the Aldosterone-Induced Ca^2+^ Channel Upregulation

**Figure 5:**
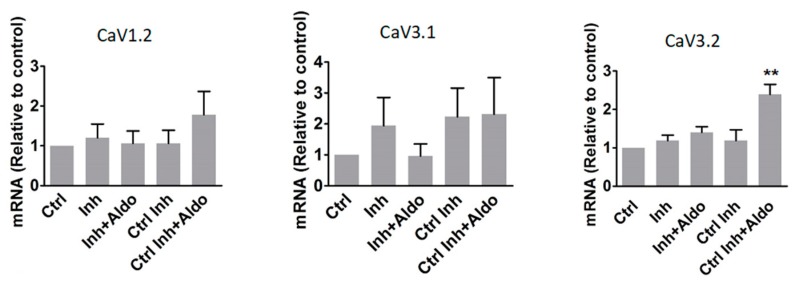
Inhibition of miR-204 expression prevents aldosterone-induced increase of calcium channel α1 subunits. Graphs show mRNA levels for L-type and T-type Ca^2+^ channel pore subunits—α1C, α1G, and α1H—in control cells (Ctrl), as well as in antagomiR-204 inhibitor (37.5 nM for 24 h)-transfected (Inh), antgomiR-204 inhibitor (37.5 nM for 24 h)-transfected + aldosterone (1 µM for 24 h)-stimulated (Inh+Aldo), luciferase siRNA (37.5 nM for 24 h)-transfected (Ctrl Inh), and luciferase siRNA (37.5 nM for 24 h)-transfected + aldosterone (1 µM for 24 h)-stimulated (Ctrl Inh + Aldo) cardiomyocytes. The bars and error bars indicate the means ± SEMs (*n* = 3). ** means *p* < 0.01 vs. control.

In order to confirm that miR-204 expression is necessary to allow aldosterone to stimulate L-type and T-type Ca^2+^ channel upregulation, we used an antisense RNA to specifically target miR-204 (named antagomir-204). Neonatal rat cardiomyocytes were transfected with antagomir-204 to block miR-204 expression induced by the hormone, while a control antisense RNA was used in control cells. After 24 h of transfection, cardiomyocytes were stimulated with aldosterone (1 µM) for an additional period of 24 h. The mRNA levels of L-type and both T-type Ca^2+^ channel pore subunits, CaV1.2, CaV3.1, and, CaV3.2, were then measured by qPCR (Figure 5). Aldosterone (1 µM for 24 h) induced a significant increase in CaV3.2 mRNA levels in cells pretreated (37.5 nM for 24 h) with the control antisense RNA (Ctrl Ihn + Aldo) but this response was almost completely prevented in antagomiR-204-treated (37.5 nM for 24 h) cardiomyocytes (Inh + Aldo). The mRNA levels of CaV1.2 and CaV3.1 were slightly increased by aldosterone in control cells, without reaching statistical significance, and this increase was prevented when cardiomyocytes were treated with antagomir-204.

**Figure 6:**
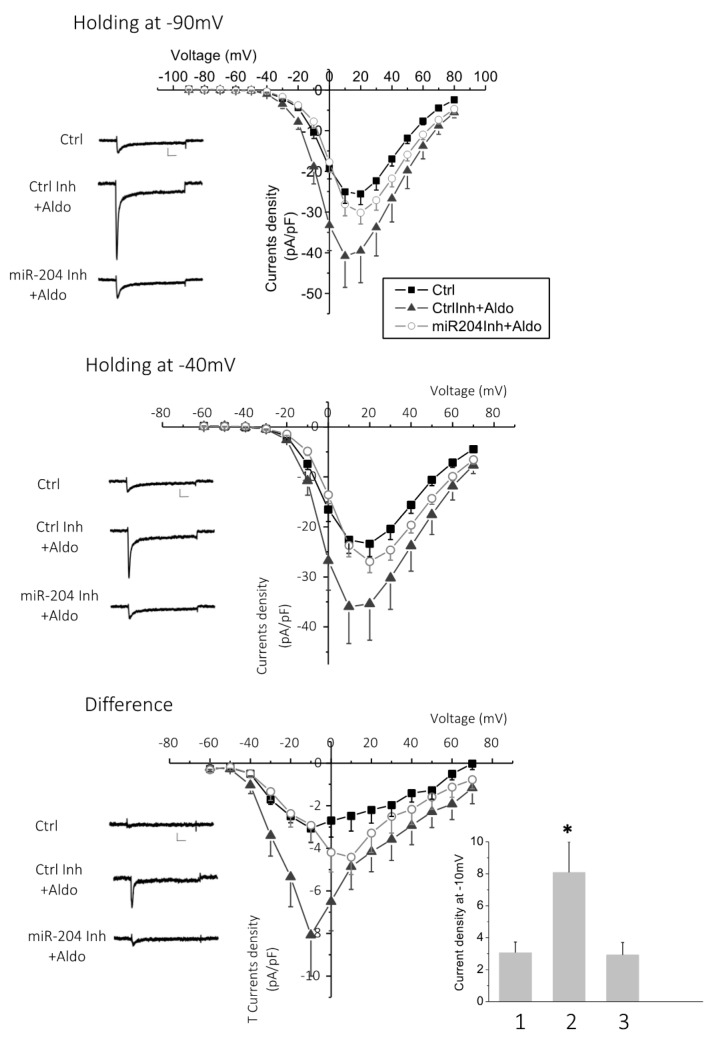
Inhibition of miR-204 expression prevents an aldosterone-induced increase of Ca^2+^ currents. Calcium currents were recorded in the whole cell configuration of the patch clamp technique in cardiomyocytes transfected (37.5 nM for 24 h) either with the antagomir targeting miR-204 (miR204Inh + Aldo) or with luciferase siRNA (Ctrl Inh + Aldo) and stimulated for 24 h with aldosterone (1 µM). Control cells (Ctrl) were naive (non-transfected) and resting (unstimulated) cardiomyocytes. The graphs show the current density–voltage relationship recorded from a holding potential at −90 mV (upper panel), reflecting the total (L-type and T-type) calcium current, or at −40 mV (middle panel), reflecting the L-type current. The difference plots that specifically reflect T-type Ca^2+^ currents are shown in the lower panel. The traces shown on the left are representative examples of recordings at −10 mV depolarization steps. Data are means + SEMs from 15 (Ctrl) or 12 (Ctrl Inh or miR204Ihn) independent cells. The bar graph represents the means and SEMs of T current density recorded at −10 mV in control cells (**1**), control inhibitor plus aldosterone (**2**), and antagomir targeting miR-204 plus aldosterone (**3**) (* *p* < 0.05).

Next, we tested whether the increase in aldosterone-stimulated T-type Ca^2+^ currents was also affected by miR-204 inhibition (Figure 6). Aldosterone (1 µM for 24 h) induced a significant increase in L-type and T-type Ca^2+^current densities in cardiomyocytes pretreated with a control antisense RNA oligonucleotide (37.5 nM for 24 h). In contrast, in antagomir-204-pretreated (37.5 nM for 24 h) cardiomyocytes, aldosterone-induced stimulation was prevented on both L-type and T-type Ca^2+^currents (Figure 6). Cell pretreatment with antagomiR-204 or the control antisense RNA, in the absence of aldosterone, had no effect on the basal L-type and T-type current densities (Figure S2).

These results thus demonstrate that miR-204 is required for the aldosterone-stimulated expression of the T-type Ca^2+^ channels pore subunit CaV3.2.

### 2.5. MicroRNA-204 Mimics the Aldosterone-Induced Downregulation of NRSF

**Figure 7:**
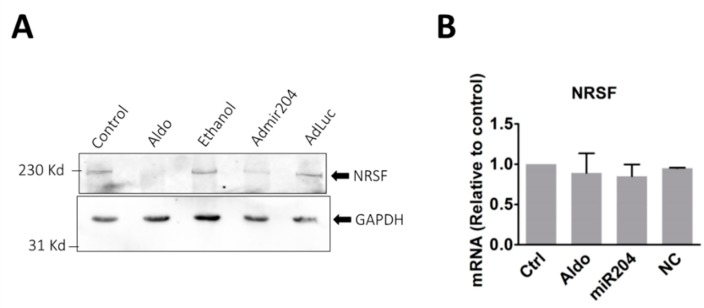
Aldosterone stimulation and miR-204 overexpression reduce REST-NRSF protein expression. (**A**) The pictures represent immunoblotting which shows the expression of REST-NRSF (upper panel) and GAPDH (lower panel) detected with specific antibodies. Cardiomyocytes were incubated for 24 h with either 1 µM aldosterone (Aldo) or ethanol (1 µL), or infected with miR-204 adenovirus (AdmiR204), or shLuc adenovirus (AdLuc) for the same period. The picture is representative of three experiments performed from three different cell preparations. (**B**) The bar graph shows the mRNA expression level of the transcriptional repressor NRSF determined by RT-qPCR in control, aldosterone (1 µM), miR204 adenovirus infected or shLuc adenovirus infected cardiomyocytes. The bars and error bars indicate the means + SEMs (*n* = 3).

Because miRNAs promote the downregulation of their specific targets [23], we hypothesized that in order to promote increased T-channel expression upon aldosterone stimulation, miRNA204 must downregulate the expression of one or several negative transcriptional regulators of T-channels that are already expressed in resting cells and therefore prevent channel expression. REST-NRSF is a known transcriptional repressor that blocks the transcription and expression of the CaV3.2 T-type channel in cardiac cells [29]. We therefore tested whether REST-NRSF protein expression was affected by miR-204 (Figure 7A). Aldosterone (1 µM) stimulation resulted in a marked reduction of REST-NRSF expression. Similarly, cardiomyocytes overexpressing miR-204 also showed a reduced expression of the REST-NRSF protein. Interestingly, neither aldosterone or miR-204 affected the messenger RNA coding for REST-NRSF, a signature of protein translation regulation by miRNA (Figure 7B).

These results thus strongly suggest that miR-204 targets REST-NRSF and reduces its expression, an effect resulting in the release of the chronic transcriptional repression of CaV3.2 by this protein.

## 3. Materials and Methods

### 3.1. Experimental Animals

The care and handling of rats for the present study were in conformity with the Guide for the Care and Use of Laboratory Animals published by the NIH (publication no. 85-23, 1996) and approved by the Animal Care and Use Committees of Nagoya University (Elucidating the mechnisms of gene expression in cardiac diseases, 2018031374, 30 March 2018).

### 3.2. Cell Culture, Stimulation, and Transfection

The hearts from sacrificed Wistar/ST three-day-old neonatal rats were removed after dissection. To isolate cardiomyocytes, cardiac muscle tissue was digested in 10 mL phosphate-buffered saline (PBS) containing 0.12% type 2 collagenase at 37 °C and pelleted by centrifugation at 950 rpm for 5 min. The cell pellet was then suspended in Dulbecco’s modified Eagle’s medium with 10% fetal bovine serum, 1% penicillin-streptomycin, and 100 nM 5-bromodeoxyuridine (BrDU) and washed five times. Cells were then plated on 10 cm dishes for 1 h to separate cardiomyocytes from fibroblasts. Finally, purified cardiomyocytes were cultured in Dulbecco’s modified Eagle’s medium with 10% fetal bovine serum, 1% penicillin-streptomycin, and 100 nM BrDU in 5% CO_2_ at 37 °C for 24 h. The culture media was changed to Dulbecco’s modified Eagle’s medium containing 1% penicillin-streptomycin, 100 nM BrDU, 1 µg/mL insulin, and 5 µg/mL transferrin.

After 24 h, cells were stimulated with 1 µM aldosterone and/or 10 µM spironolactone, infected with miR-204 adenovirus and short hairpins targeting luciferase (shLuc) adenovirus, or transfected with miR-204 inhibitor, luciferase siRNA, and 40 ng of plasmids containing GFP (Green Fluorescent Protein) using Lipofectamine 3000. Adenovirus for human miR-204 was purchased from ViGene (Rockville, MD, USA). Recombinant human adenoviruses-5 encoding shLuc were constructed, amplified, and purified as previously described [11]. MiScript miRNA inhibitor was obtained from QIAGEN (Hilden, Germany). Luciferase siRNA (sense strand, 5′-CUUACGCUGAGUACUUCGATT-3′; antisense strand, 5′-UCGAAGUACUCAGCGUAAGTT-3′; where lower cases indicate DNA) was purchased from Gene Design Inc. (Osaka, Japan).

### 3.3. MicroRNA Preparation and Screening by Microarray

Total RNA was prepared using Trizol (Invitrogen, Carlsbad, CA, USA), and miRNA were isolated using an RNeasy MinElute CLEAN UP kit (Qiagen) or a Mirvana kit (Thermo Fisher Waltham, MA, USA). Global miRNA expression was then determined using TaqMan Low Density Arrays (TLDA) (Applied Biosystems, Foster, CA, USA), according to the manufacturer’s protocols on an AB7900 platform.

### 3.4. RNA Isolation and Real-Time Quantitative Reverse Transcription (RT)-Polymerase Chain Reaction (PCR)

Total RNA from cardiomyocytes was extracted using TRIreagent (Sigma-Aldrich, St. Louis, MO, USA) according to the manufacturer’s indications. Total RNA was reverse transcribed using ReverTra Ace qPCR RT Kit (Toyobo, Osaka, Japan). Real-time quantitative PCR was performed with the synthesized cDNA (50 ng of RNA contents), 250 nM primers, and THUNDERBIRD SYBR qPCR Mix (Toyobo, Osaka, Japan) at a final volume of 25 µL. Stratagene Mx3000P (Agilent Technologies, Santa Clara, CA, USA) was used for the PCR reaction. The mRNA levels were normalized to those coding for glyceraldehyde 3-phosphate dehydrogenase (GAPDH). The primer sequences are indicated in Table 1.

MicroRNAs from cardiomyocytes and isolated mice hearts were extracted using mirVana miRNA Isolation Kit (Ambion, Thermo Fisher, Waltham, MA, USA) according to the manufacturer’s indications. cDNAs were synthesized with 10 ng of total miRNAs using the Taqman miRNA reverse transcription kit (Applied Biosystems, Foster City, CA, USA). Real-time quantitative PCR was performed with synthesized cDNA (0.89 ng of total RNA contents), Taqman small RNA assay and Taqman universal PCR Master MixII (Applied Biosystems, Foster City, CA, USA) in a final volume of 20 µL. A 7300 real-time PCR system (Applied Biosystems, Foster City, CA, USA) was used for the PCR reaction. The miR204 levels were normalized to U6 SnRNA.

### 3.5. Protein Isolation and Western Blot

Whole cell proteins were isolated from neonatal cardiomyocytes using an ice-cold RIPA buffer (50 mM Tris-HCL, 150 mM NaCl, 5 mM EDTA, 1% Triton, 0.5% Na-deoxicolate) supplemented with complete protease inhibitor from (Roche, Basel, Switzerland). Protein concentrations were determined by BCA assay (Thermo Fisher Scientific, Walmath, MA, USA). Twenty micrograms of protein was loaded into a 7.5% SDS-polyacrylamide gel and separated by electrophoresis. Proteins were then transferred on polyvinylidene fluoride (PVDF) membrane. The PVDF membrane was immunoblotted with a rabbit polyclonal antibody targeting RESF-NRSF (1/1000) (Cell Signaling, Danvers, MA, USA) and a monoclonal antibody targeting GAPDH linked with a horseradish peroxidase (HRP) (Cell Signaling, Danvers, MA, USA) for detection. To detect NRSF, an anti-rabbit IgG coupled with HRP (GE Healthcare, Chicago, IL, USA) was used. Chemiluminescence was detected using an ECL detection kit (Millipore Corp., Burlington, MA, USA), and images were captured with a cooled-CCD camera of a LAS-1000 plus luminescent image analyzer (Fujifilm, Tokyo, Japan).

### 3.6. Patch Clamp

Cardiomyocytes plated on poly-l-lysine coated glass bottom dishes stimulated by aldosterone (1 µM), in the presence or absence of spironolactone (10 µM) and infected with adenovirus for miR-204 or shLuc, or transfected with miR-204 inhibitor, luciferase siRNA, and GFP plasmids.

Patch clamp micropipettes from borosilicate glass capillaries with 0.86 mm diameters (Harvard apparatus, Holliston, MA, USA) were fabricated using a puller PC-10 (Narishige, Tokyo, Japan). The snap of the micropipette tip was fire polished using a microforge MF-900 (Narishige, Tokyo, Japan). Cells were placed in a bath solution containing (in mM) 125 *N*-methyl-d-glucamine, 5 4-aminopyridine, 20 tetraethylammonium chloride (TEA Cl), 2 CaCl_2_, 10 d -glucose, and 10 HEPES (pH 7.4 with TEAOH). A dish was placed on an inverted microscope Olympus IX71 (Olympus, Tokyo, Japan). The micropipette was filled with a pipette solution containing (in mM) 130 CsCl, 10 EGTA, 25 HEPES, 3 Mg-ATP, and 0.4 Li-GTP (pH 7.2 adjusted with CsOH).

After formation of the whole-cell patch clamp configuration, Ca^2+^ currents were measured in the voltage-clamp mode using an Axopatch 200B amplifier (Molecular Devices, St. Jose, CA, USA). The cell was held at a membrane potential of −80 mV and depolarized by 10 mV voltage steps up to +80 mV to measure T- and L-type Ca^2+^ currents. Then, the cell was held at a membrane potential of −40 mV and depolarized by 10 mV voltage steps from −60 mV to 70 mV to measure isolated L-type currents (after T channel inactivation). The leak was subtracted automatically by Clamp 10 software (Molecular Devices, St. Jose, CA, USA).

### 3.7. Data Analysis

Statistical analysis was performed by one-way ANOVA followed with the Turkey–Kramer’s test or by unpaired Student’s *t*-tests. Data are presented as means ± SEMs (* means *p* < 0.05, ** *p* < 0.01, and *** *p* < 0.001 compared to control samples).

## 4. Discussion

We found here that miR-204 is the main microRNA upregulated by aldosterone in cardiomyocytes. The increase of miR-204 expression occurs downstream of MR activation. In turn, our results suggest that miR-204 contributes to the increased expression levels of T-type Ca^2+^ channels and, therefore, to the spontaneous cardiomyocyte beating frequency. In the heart, multiple microRNAs regulate the expression of ion channels that contribute to the development of cardiac pathologies, such as arrhythmias and atrial fibrillation. MicroRNAs can directly target ion channel transcripts and downregulate their expression. The HCN4 channel is a target of miR-423-5p in the heart sinus node [30]. Both miR-328 and miR-21 were found to downregulate *CANA1C* and *CACNB2* genes, which encoding, respectively, for the pore and for an auxiliary subunit of the L-type voltage-gated Ca^2+^ channel [31,32]. In contrast, miR-219 promotes increased expression of *SCN5A*, encoding for the pore subunit of the voltage-gated sodium channel, probably through indirect targeting of the *SCN5A* transcripts [33]. Our results show that miR-204 promotes the increased expression of both *CACNA1G* and *CACNA1H* mRNA, and, in this way, upregulates T-type Ca^2+^ currents. A similar effect has been observed for *CACNA1C* and L-type currents, although to a lesser extent. Therefore, miR-204 apparently plays a role downstream of MR activation for the re-expression of T-type channels. We hypothesized that miR-204 does not directly target T-channel encoding genes, but probably, some putative protein that negatively regulates channel expression. Recent studies have shown that miR-204 regulates the expression of transcription factors, such as forkhead box C1 (FOXC1) and myeloid ecotropic viral integration site 1 homolog 2 (MEIS2), which are both transcription factors involved in the eye development [34]. In pancreatic beta cells, miR-204 targets the insulin transcription factor MAFA [35]. The increased expression of T-type calcium channel in response to miR-204 suggests that the miRNA targets some intermediate negative transcriptional regulator. Indeed, our results show that aldosterone and miR-204 overexpression inhibit the expression of REST-NRSF protein, a transcriptional repressor known to target CaV3.2 expression in cardiomyocytes [29]. This protein thus appears to be a relevant candidate for linking miR-204 increase and T-channel upregulation. However, the possibility that other transcriptional regulators, also playing a role in T-channel upregulation, might be targeted by miR-204 remains to be tested.

In a recent study performed in vivo, aldosterone was found to stimulate the expression of multiple miRNAs, miR-21 being the most upregulated in the left ventricle of the heart [36]. This difference from our present study might be due to the duration of aldosterone stimulation and the presence of other cell types in the left ventricle of the heart in vivo (fibroblasts and macrophages). Indeed, in the present study, the stimulation was acute, for 3 to 24 h, whereas the stimulation performed in vivo in the study of Ball et al. was chronic, with treatments occurring over two weeks. Aldosterone might stimulate a variety of microRNAs at different times that contribute to the dynamic cardiomyocyte response.

MicroRNAs also play a role in the development of the heart. In particular, miR-204 has been shown to promote the proliferation and differentiation of human-derived cardiomyocyte progenitor cells [37]. In vitro, miR-204 also stimulates the proliferation of rat neonatal and adult cardiomyocytes. In addition, the overexpression of miR-204, specifically in the heart ventricles of mice, has been shown to lead to heart hypertrophy [38].

Our results suggest that miR-204 is upregulated downstream of MR upon aldosterone stimulation of cardiomyocytes (Figure 1B). The effect of aldosterone was prevented by spironolactone, an MR antagonist, suggesting that miR-204 upregulation is induced via MR activation in aldosterone-stimulated cardiomyocytes. We cannot exclude the involvement of additional receptors, in particular, the glucocorticoid receptor. Further studies are required to confirm the exclusivity of MR in the activation of miR-204. It would be also worthwhile to determine whether the miR-204 promoter, located on *TRPM3* (*Transient Pore Receptor Member 3*) gene [39], has specific steroid hormone-response elements, which MR binds to for the regulation of gene transcription [40].

In conclusion, our study strongly suggests that miR-204 expression in ventricular cardiomyocytes is necessary for aldosterone-induced T-channel upregulation, leading to an increase in low voltage-activated Ca^2+^ currents and therefore, to the acceleration of ventricular cell spontaneous beatings. Because this chronotropic action of aldosterone is recognized as a mechanism that possibly leads to ventricular electrical dysfunction, targeting miR-204 could provide new insights into the prevention of cardiac arrhythmia and heart failure.

## Figures and Tables

**Table 1:** Primers Used in Real-Time Quantitative PCR.

Gene	Oligonucleotide Sequence
*α1C*	Fw: 5′-AGCAACTTCCCTCAGACGTTTG
	Rev: 5′-GCTTCTCATGGGACGGTGAT
*α1G*	Fw: 5′-ACGCTGAGTCTCTCTGGTTTGTC
	Rev: 5′-TGCTTACGTGGGACTTTTCAGA
*α1H*	Fw: 5′-GGCGAAGAAGGCAAAGATGA
	Rev: 5′-GCGTGACACTGGGCATGTT
*NRSF*	Fw: 5′-GGCCAAACCCTTCCGTTGT
	Rev: 5′- TGGCTTGCTTCTCTGCACT
*GAPDH*	Fw: 5′-CAACTCCCTCAAGATTGTCAGCAA
	Rev: 5′-GGCATGGACTGTGGTCATGA

The table shows the sequences of the primer pairs used for the real-time quantitative PCR experiments in the present study. Fw: forward primer Rev: reverse primer.

## Supplementary Materials

It can be found in the following link: http://www.mdpi.com/1422-0067/19/10/2941/s1.
