# Supplementary material for: MicroRNA-204 Is Necessary for Aldosterone-Stimulated T-Type Calcium Channel Expression in Cardiomyocytes
Source: Int J Mol Sci. 2018 Sep 27;19(10):2941. doi: 10.3390/ijms19102941 (PMC6212903; doi:10.3390/ijms19102941)
Supplement: Supplementary file 1 [file ijms-19-02941-s001.pdf]

Supplementary Materials

A

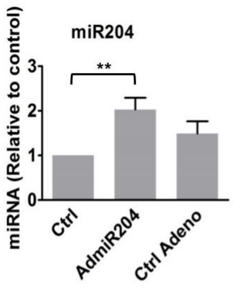

B

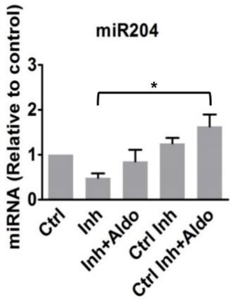

Figure S1. Expression levels of miR-204 in neonatal cardiomyocytes upon overexpression or repression. (**A**) Graphs show miR-204 levels in control, miR-204 adenovirus-infected (AdmiR204), or shLuc adenovirus-infected (Ctrl Adeno) cardiomyocytes. Bars and error bars indicate the mean ± SEM (n = 3). ** means $p<0.01$ vs control. (**B**) Graphs show miR-204 levels in control (Ctrl), antagomiR-204 inhibitor (37.5 nM for 24 h)-transfected (Inh), antagomiR-204 inhibitor (37.5 nM for 24 h)-transfected + aldosterone (1 μM for 24 h)-stimulated (Inh+Aldo), luciferase siRNA (37.5 nM for 24 h)-transfected (Ctrl Inh) or luciferase siRNA (37.5 nM for 24 h)-transfected + aldosterone (1 μM for 24 h)-stimulated (Ctrl Inh+Aldo) cardiomyocytes. Bars and error bars indicate the mean ± SEM (n = 3). * means $p < 0.05$ vs control.

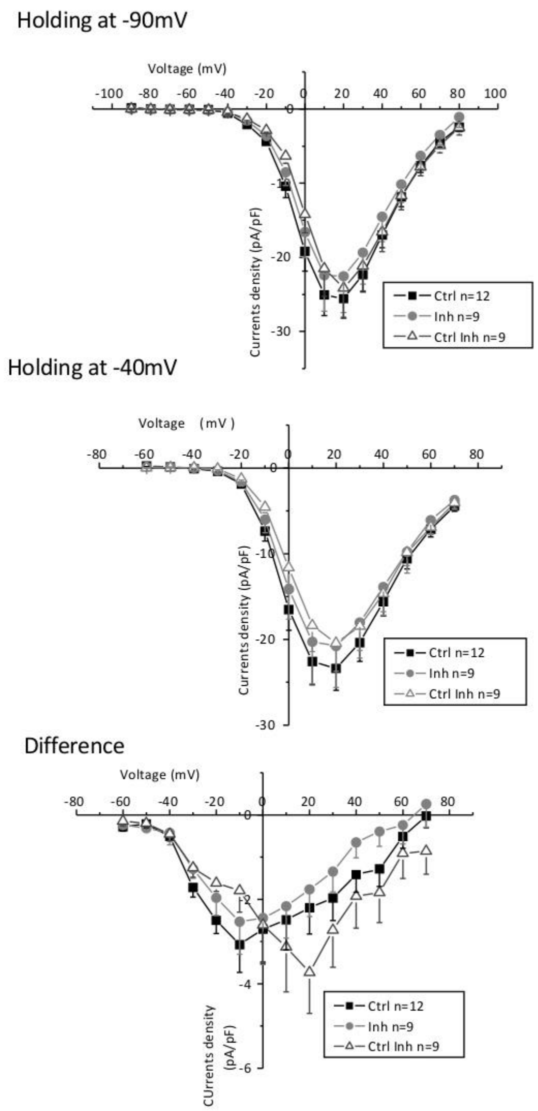

Figure S2. The presence of the miR-204 antagomir alone does not affect resting calcium currents. Cardiomyocytes were transfected (37.5 nM for 24 h) with either the antagomir targeting miR-204 (Inh) or with luciferase siRNA (Ctrl Inh). Control cells were naïve, non-transfected cells. Graphs show IV relationship in control and transfected cells recorded at various voltages from a holding potential at -90 mV (upper panel) or -40 mV (middle panel). The difference plots reflecting T-type Ca^{2+} currents are shown in the lower panel. Data are mean ± SEM from 12 (Ctrl) or 9 (transfected) independent cells.
